# The impact of Hurricane Maria on Puerto Rico’s health system: post-disaster perceptions and experiences of health care providers and administrators

**DOI:** 10.1186/s41256-021-00228-w

**Published:** 2021-11-10

**Authors:** Sheilla L. Rodríguez-Madera, Nelson Varas-Díaz, Mark Padilla, Kevin Grove, Kariela Rivera-Bustelo, Jeffrey Ramos, Violeta Contreras-Ramirez, Sergio Rivera-Rodríguez, Ricardo Vargas-Molina, Jose Santini

**Affiliations:** 1 Department of Global and Sociocultural Studies, School of International and Public Affairs, Florida International University, 11200 SW 8th Street, SIPA #334, Miami, FL 33199 USA; 2 Social Sciences Department, Medical Sciences Campus, University of Puerto Rico (UPR), San Juan, PR, USA; 3 School of Behavioral and Brain Sciences, Ponce Health Science University, Ponce, PR, USA; 4 Department of Community Health and Health Policy, City University of New York, New York, USA; 5 Center for Evaluation and Sociomedical Sciences, Medical Sciences Campus, UPR, San Juan, PR, USA; 6 Health and Acupuncture for the People Project, PR, USA

**Keywords:** Health care system, Post-disaster, Health care providers, Resilience, Puerto Rico

## Abstract

**Background:**

After its landfall in Puerto Rico in 2017, Hurricane Maria caused the longest blackout in United States history, producing cascading effects on a health care system that had already been weakened by decades of public sector austerity and neoliberal health reforms. This article addresses how health care professionals and administrators experienced the health care system’s collapse and the strategies used by them to meet their communities' health needs.

**Methods:**

Data were collected between September 2018 and February 2020. Ethnographic observations in health care facilities and semi-structured qualitative interviews with representatives of the health care system were conducted. This paper focuses on data from interviews with health care providers (n = 10) and administrators (n = 10), and an ethnographic visit to a pop-up community clinic. The analysis consisted of systematic thematic coding of the interview transcripts and ethnographic field notes.

**Results:**

Results provide insight on how participants, who witnessed first-hand the collapse of Puerto Rico’s health care system, responded to the crisis after Maria. The prolonged power outage and lack of a disaster management plan were partly responsible for the death of 3,052 individuals who experienced extended interruptions in access to medical care. Participants reported a sense of abandonment by the government and feelings of mistrust. They also described the health sector as chaotic and lacking clear guidelines on how to provide services or cope with personal crises while working under extreme conditions. In such circumstances, they developed resilient responses to meet communities’ health needs (e.g., itinerant acupuncture services, re-locating physicians to local pharmacies).

**Conclusions:**

Participants’ narratives emphasize that the management of Hurricane Maria was fraught with political and economic constraints affecting Puerto Rico. Ineffective planning and post-Maria responses of the local and federal governments were determinants of the disaster’s impact. The findings contribute to a growing scientific literature indicating that Hurricane Maria revealed ‘the collapse before the collapse,’ alluding to the structural deficiencies that presaged the catastrophic event. In the context of governmental abandonment, the authors argue for the importance of developing alternative strategies in post-disaster health care provision among health professionals and administrators who work at the front lines of recovery.

**Supplementary Information:**

The online version contains supplementary material available at 10.1186/s41256-021-00228-w.

## Background

Puerto Rico, a Caribbean island with a Spanish-speaking population of 3.5 million [1], has been a United States (US) territory since it was ceded by Spain in 1898 [2]. Since then, Puerto Rico has been marked by its colonial status, which has contributed to a history of systematic discrimination and exclusion in relation to the US. For example, Puerto Ricans, although US citizens, lack key rights enjoyed by citizens in the continental US. They cannot cast electoral votes in general presidential elections; are subjected to federal laws but lack voting representation in Congress; and receive fewer federal governmental benefits than other Americans, including those aimed to improve health care access [2]. Still, Puerto Ricans contribute economically to national health programs, including Social Security and Medicare, to which they have reduced access due to mandated restrictions on these benefits for residents on the Island [3]. During the past decades, Puerto Rico’s economy has languished following the elimination of US-based tax protections, the privatization of critical infrastructure as part of neoliberal reforms (including the health care system), the accumulation of a $70 billion external debt without the right to bankruptcy, and local government mismanagement. It is against this colonial backdrop that Puerto Rico has suffered from the catastrophic effects of natural disasters [4–8].

The US Congress has imposed several neoliberal policies on Puerto Rico that are directly relevant as background to the current analysis [8]. First, in response to the fiscal crisis, the Puerto Rico Oversight, Management, and Economic Stability Act (PROMESA) was signed into law in 2016. Based on the fact that Puerto Rico, as a territory, could not access federal protections from Washington, D.C. to cope with its uncontrolled debt, this law established an unelected oversight board (often called ‘*la Junta*’ locally) to assist the Puerto Rican government in managing its finances and ensuring debt repayment. *La Junta* then mandated austerity measures, such as cutting retirees’ pensions and closing public schools, severely impacting daily life. Some of the most devastating effects have been in the public health sector [9]. One year after PROMESA was passed, and in the midst of political tensions resulting from the financial crisis, Hurricane Maria hit the Island. This category 4 event was the most massive natural disaster to ever affect Puerto Rico [10], significantly damaging its electrical grid [11]. The storm caused the longest blackout in US history, and the second-longest globally, taking almost 11 months to fully restore power [12, 13]. The outage affected 100% of the population and had cascading effects on multiple areas, including the functioning of the health care system [14].

The published literature addressing the effects of Hurricane Maria on the Island has exposed the severe vulnerabilities of its health care system [15–19], including lethal gaps in access to medication by patients with chronic diseases (e.g., renal disease, diabetes, respiratory diseases) [11, 20–24] and the interruption of life-sustaining treatments (e.g., dialysis, chemotherapy) [3, 15, 16]. In fact, these failures were partly responsible for the more than 3000 deaths ascribed to the natural disaster [25]. Furthermore, existing publications on the matter agree on one central issue: the devastating aftermath of this natural event exacerbated an already fragile health care infrastructure [2, 3, 7–10, 12–16, 19]. This study aims to understand health care providers’ and administrators’ perceptions of Hurricane Maria’s impact on the Puerto Rico’s health care system and their experiences in the provision of medical care in a post-disaster setting.

## Methods

The present article’s aims derive from an NIH-funded study (1R21AG063453) that systematically documented the challenges faced by the Puerto Rico’s health care system during and following Hurricane Maria while also exploring dimensions of health sector resilience.

### Research design/framework

The research team implemented an exploratory-descriptive research design using a qualitative methodological approach that integrated ethnographic observations and semi-structured interviews (SSI). The study was informed by the Framework for Healthcare Disaster Resilience (FHDR). This framework was developed by the Center for Health Security at John Hopkins’s Bloomberg School of Public Health [26] in order to foster post-disaster resilience in the US’s health care system based on an analysis of potential threats, existing gaps, and community resources. The FHDR posits that: (1) the US is not well prepared for large-scale natural disasters (e.g., Hurricanes Maria, Sandy, Katrina; moderate earthquakes) or catastrophic health events (e.g., COVID-19) and should therefore incorporate disaster resilience models into planning; (2) chronically ill people are among the most vulnerable to disaster-related events, and should be prioritized in research and planning; and (3) evidence-based initiatives should be designed to foster health sector resilience, particularly to meet the urgent medical needs of the most vulnerable populations during and after disasters. These initiatives should include strategies to promote a ‘culture of resilience’ among grassroot and community-based organizations to enable robust healthcare coalitions capable of responding effectively to disasters [26].

### Participants

The study involved interviews with a diverse sample of representatives from three key levels of the health care system: the institutional level (policy makers), the administrative level (health facility administrators), and the practical level (health providers and patients). This paper focuses specifically on data gathered from health care providers and administrators to understand their perceptions of Hurricane Maria’s impact on the Puerto Rico’s health care system and their experiences in providing medical care in a post-disaster setting. The data were drawn from two main sources: (a) ethnographic observations and (b) SSI with 67 representatives of three levels of the health care system. SSI participants were recruited using a purposive sampling strategy. Purposive sampling strategies are based on the assumption that, given the aims of the study, specific kinds of people may hold different and important views about the ideas and issues in question and therefore should be included in the sample [27]. In this case, this sampling approach was employed to ensure the diversity of representatives of the three levels of the health care system. Furthermore, the recruitment process was balanced in terms of urban and rural geographies in 12 municipalities of Puerto Rico (San Juan [n = 9], Ponce [n = 2], Bayamón [n = 2], Caguas [n = 1], Guaynabo [n = 1], Vieques [n = 1], Corozal [n = 1], Yabucoa [n = 1], Barranquitas [n = 1], and Cabo Rojo [n = 1]), thus allowing the research team to understand the experiences of participants throughout the Island.

The selection of participants was based on two inclusion criteria: (1) be 21 years of age or older (the age of adulthood in Puerto Rico); and (2) having been engaged in one of the three levels of the health care system during the catastrophic event. The research team terminated the recruitment of participants at 67 since the preliminary qualitative data analysis evidenced thematic saturation [28]. This paper focuses specifically on a total of 20 SSI with 10 health care providers (5 from rural and 5 from urban areas) from diverse disciplines (e.g., physicians, nurses, psychologists), and 10 administrators (5 from rural and 5 from urban areas) from both governmental and private facilities. Concentrating on this segment of the sample allowed the team to describe how these health care providers and administrators experienced the challenges posed by Hurricane Maria and to identify dimensions of resilience in their responses.

### Instruments

The research team developed an SSI guide in Spanish, which included 38 questions divided into four domains: (1) sociodemographic information, (2) significant challenges for the health care delivery system during and after the event, (3) resilience strategies implemented in health care provision, and (4) needed changes for managing future catastrophic events at each level (i.e., institutional, administrative and practical). The sociodemographic section gathered information regarding participants' age, civil status, and income, among other characteristics. The section on major challenges faced by the health care system included questions intended to explore participants’ perceptions about those challenges (e.g., why they happened, how were they experienced, where they took place, and for how long they were manifested) and their personal experiences during and after the event. The third section included questions about participants’ perceptions regarding dimensions of resilience expressed as part of health care provision during and after Hurricane Maria (e.g., the role of the local and federal governments, patients, and communities in health sector resilience). The last section included questions to elicit participants’ opinions and recommendations on the changes needed to prepare Puerto Rico for a similar catastrophic event in the near future. This guide allowed the team to uniformly ask the same set of questions to all participants while simultaneously allowing flexibility to pursue new topics as needed.

In addition, the research team developed an Observation Guide to systematically observe the geographic, social, and institutional qualities through ethnographic observations (e.g., the physical space where the health care service was provided, the environment, and social interactions between health care providers, staff members, and patients).

### Procedure

The study lasted 18 months (September 2018 and February 2020) and included two phases. The first phase focused on ethnographic observations, while the second involved the SSI. Once the investigators obtained authorization from the University of Puerto Rico’s Medical Sciences Campus’s Institutional Review Board (Protocol Approval #A9980218), the team commenced the ethnographic phase (from January to June 2019). Ethnographic observations were conducted in eight health care provision sites (e.g., hospitals, pop-up clinics, community-based organizations) in rural and urban settings throughout the Island. These sites were purposively selected to account for the diverse health service provision contexts (e.g., rural vs. urban; governmental vs. private) that were active during and after Hurricane Maria. During the ethnography, the team had the opportunity to informally interact with health care providers, administrators, support staff, and community members. Extensive field notes were taken by the ethnographers.

Three months after starting the ethnography, the SSI phase began (from April to December 2019). The recruitment process for the SSI included two mechanisms for identifying potential participants: (a) through contacts made during the ethnographic observations and (b) via the web pages of hospitals and private medical offices. Since the ethnographic process allowed the researchers to interact with multiple actors from the different levels of the health care system, members of the research team began to approach individuals during the site visits to participate in the SSI by selecting and screening potential participants during observation sessions. In addition, the team proceeded to contact individuals through phone calls to offices using contact information gathered from professional websites. In both cases, the team explained the nature of the study and asked about their interest in participating. Those who agreed to participate were invited to meet for informed consent and interview. SSI took place in private locations convenient for participants. These included organizational and private offices and university campuses. The study’s aims, structure, and confidentiality protocol were explained to the participants (including the use of pseudonyms in all publications), who then gave their written consent to participate. All SSI were conducted in person, except one that was carried out via Skype. Interviews lasted an average of 2 hours. Participants did not receive compensation for their participation.

### Analysis

All SSI were audio-recorded and transcribed by the research team, and each transcript was assigned a unique identification code and a pseudonym. The analysis consisted of a systematic thematic coding [29] of the interview transcripts and ethnographic field notes using a two-staged technique [30, 31]. In the first stage, open coding of all transcripts and field notes regarding themes related to the analytic foci were conducted (e.g., the effects of Hurricane Maria on the health care system, manifestations of challenges and resilience in health care provision). This allowed for the understanding of participants’ perceptions and experiences, and how these were embedded in the social and structural challenges faced by the Island before, during and after the event. Throughout the coding process, analytic memos were written to refine interpretations. During a subsequent stage, each investigator/coder wrote analytic summaries of the transcripts, which were then brought back to the investigative team meetings for group reflection and analytical discussions. These discussions continued until all coders agreed on the interpretation of selected texts and consensus was reached. The consensus was established through an analytic process of discussing similarities and differences in interpretation; differences in interpretation then contributed to the refinement of the coding scheme or informed the analytic summaries. The consensus was then incorporated into an NVivo database. For this paper, a horizontal review of all analytic summaries was implemented to extract and refine core themes and contextualize them within the participant’s larger narrative.

## Results

### Demographic characteristics of the sample

Most participants were female and between the ages of 26 and 75. The sample was equally distributed in terms of marital status and geographical area in which they provided health care services. Table 1 includes basic sociodemographic characteristics of the health care providers and administrators who participated in the SSI sample.

**Table 1:** Sociodemographic characteristics of health care system professionals

Baseline characteristics	Health professional^a^		Health facility administrators	
	n	%	n	%
*Gender*				
Female	7	70	6	60
Male	3	30	4	40
*Age*				
18–25	0	0	0	0
26–35	2	20	0	0
36–45	3	30	0	0
46–55	3	30	4	40
56–65	2	20	4	40
66–75	0	0	2	20
> 75	0	0	0	0
*Marital status*				
Single	6	60	2	20
Married	2	20	6	60
Consensual union	2	20	1	10
Divorced	0	0	1	10
*Work*				
Urban area	4	40	6	60
Rural area	4	40	4	40
Both area	2	20	0	0
*Monthly income*				
Not available	0	0	1	10
$1,800.00–$3,000.00	3	30	3	30
$3,001.00–$5,000.00	4	40	4	40
$5,001.00–$7,000.00	1	10	1	10
$7,001.00–$9,000.00	1	10	0	0
$9,001.00–$10,000.00	0	0	0	0
$10,001.00–$12,000.00	0	0	1	10
$12,001.00–$15,000.00	1	10	0	0

*n* = 20 (*n* = 10 for each category)

^a^Health professionals include physicians, nutritionists, nurses, psychologists, social workers, academics, and researchers in the natural and social sciences

The results presented here were organized into three themes identified in the analysis: (a) experiences of the collapse of the health care system, (b) a sense of abandonment of the health care sector by policy makers during the disaster response, and (c) challenges and resilience in health care provision.

### The collapse of the health care system

One key finding in the analysis was the fact that many participants described the aftermath of the storm as a total “collapse” of the health care system that began prior to the event itself. “The collapse of the health care system started before the hurricane, so the hurricane was just a blowout,” observed “Orlando,” a health care provider who serves patients in urban and rural communities and had directly witnessed the aftermath of the storm. Another participant, “Martin”, a primary care physician, described the destruction of the health care system as a part of a longer, “invisible” collapse:

The collapse was in place before [Maria], and we [Puerto Ricans] made it invisible […], and what the Hurricane let you know is that there is no way you can put a tablecloth over it to cover what is happening, mainly because it is something that is prolonged.

Orlando and Martin’s narratives pointed to a deterioration of the physical infrastructure that preceded Hurricane Maria's impact on the Island. For example, all participants mentioned the fragility of the Island’s electrical grid, which made the blackout that followed Maria virtually inevitable. For example, two participants shared the following:

I think that the biggest problem lies on the power grid issue. Without electricity, the pharmacies were not working; the clinics were not working... The people who needed a breathing machine, they couldn't use it. (“Junior”, a physician)

Our electric grid was not prepared to withstand the blow that it took. It is a very old system that… I don't have to say this; everyone knows this. Even Trump himself! The grid was so old that any small event would take it out. (“Teresa”, a community-based clinic’s administrator).

There were others who emphasized that the collapse after Maria encompassed multiple sectors beyond health care, affecting every aspect of the Island’s vital infrastructure:

[It was] not merely the health [care] system… it was an entire collapse. The Island’s infrastructure collapsed. It did not exist, in all senses… Therefore, there was no government, so the health system went down the drain. (“Elena”, a psychologist)

The collapse of the Island’s basic infrastructure, and the subsequent collapse of the health care system after the event, had different implications at the individual level (for patients and providers, for example) and the structural level. “Inés”, a health care provider specializing in epidemiology, offered concrete examples of the multilevel manifestations of the collapse:

The system collapsed in terms of the access [to health care] that patients had due to transportation issues. […] Another way to look at the collapse is when you have to start reducing your services. For example, if I provide 8 hours of dialysis a day, but the power generator is running out, I have to reduce it to 4 hours, meaning fewer patients will now receive the service.

Inés continued to explain the collapse at the structural level, observing:

[…] As you go up to the structural level, it is more difficult to see the collapse, even if it is there. That happened with the government. Look, the Department of Health’s office that is dedicated to epidemiology and research has a surveillance system of notifiable diseases. That system collapsed. Why? Because that system requires that your people at the health region level continue to maintain reports so that they can monitor what is happening. With the outbreak of leptospirosis post-Maria, there was no concrete data to declare it was an epidemic issue. In truth, it turned out that [the information] was not being collected. […] In fact, we don't have data on anything that happened in those weeks [after the Hurricane], and that's an example of the collapse at the structural level.

In addition to offering examples of the myriad manifestations of collapse, most participants (17 out of 20) understood that the magnitude of the collapse was due primarily to poor disaster governance, as described below.

### Abandonment of the health care sector during the disaster response

Health care providers and administrators were among the first to detect government failures in handling the disaster that were catalysts for the collapse of the health system. They directly witnessed the chaotic governmental response to Maria, represented, on the one hand, by the absence of an agile and appropriate plan to manage or address the crisis in an efficient manner, which some participants described as “abandonment.”

Here in our clinic, until today, there has been no presence. It has been a total abandonment: neither the local government, the municipality of San Juan, nor the federal government. We know how the federal government has treated this Island, and they continue to treat it. (Teresa)

On the other hand, the disorderly response reflected not only inadequate coordination of efforts and the absence of protocols but also a lack of common sense in decision-making. As Junior expressed:

There was no coordination at all. […] And with this, they showed that the health system in Puerto Rico and health are not a priority for the government.

This same professional criticized authorities for converting the Puerto Rico Convention Center—a multi-million-dollar climate-controlled facility that comfortably housed federal disaster relief teams while the rest of the Island was abandoned in a year-long blackout—into the command site for the emergency response effort:

I think that there is ineptitude [from the government] but there is also an alienation because as long as I am going every day to this place where there is air conditioning, Internet, food, and an open bar where I can drink… well, what is the crisis? […] And meanwhile, people are dying because they do not receive medical services. (Junior)

Another example of health sector abandonment was perceived by participants in the case of the military hospital ship (USNS Comfort) that was sent to the Island 2 weeks after Hurricane Maria. “Josefina,” an oncologist who provides services in San Juan, described her anger and frustration upon discovering, in the midst of the impossibly slow response, that the ironically-named US Navy ship had visited the Island with the resignation and lack of urgency of a tourist:

There was the hospital ship that, what it did was go around the Island, and it must have seen our beautiful beaches… But did we really benefit from that? Nooo, and it was because it was not well thought out. On that ship, you were bringing some physicians who didn't speak Spanish and, on top of that, did not have a license to practice in Puerto Rico. That could be resolved quickly, but it was not fixed either. […] The government wanted to project itself as if it were the great shit. Of course, it sounds nice in terms of public relations to say, "Hell, I brought the hospital ship from the Navy, I’m the big shit. I'm the Commander in Chief,” but what the fuck?! What did we solve with that?

In general, health professionals expressed the sentiment that Puerto Rico's health care system's crisis was rooted in a longer history of political ineptitude. One of the areas where this was most evident to participants was the mismanagement of Maria's death toll estimation [25]. In the words of “Victoria”, a psychologist who works in a rural area:

There is a lot of mistrust. For an extended period, the government only recognized 64 deaths when, in reality, there were thousands. We don't believe in the state government. We don't believe in the federal government either.

The perception among health professionals that they, along with the general Puerto Rican population, had been abandoned was heightened as they personally confronted the many challenges of delivering care in the absence of meaningful disaster relief initiatives and resources.

### Challenges and resilience in health care provision

The narratives provided by health professionals and administrators regarding challenges in health care provision in Maria's aftermath were varied. In one way or another, all participants described poor work conditions, which impeded the provision of quality services. Five of them elaborated on the hospital ship USNS Comfort mentioned above, which was described as an enormous failure due to excessive bureaucratic red tape. Specifically, one health care administrator explained as follows:

We only managed to send two patients [to the ship] … The hospital ship had so many requirements in order to transfer a patient that it was impossible… So many filters. (“Ernesto”, a physician who administered a clinic in San Juan).

On this matter, “Josephina” shared:

At one point, it was said that they [administrators on USNS Comfort] did not want to admit cancer patients, they did not want to admit complicated trauma patients, and I was wondering, what are they going to admit then? Do you follow me? They [the government] filled their mouths saying the hospital ship came here, but it really didn't solve much.

As expected, the difficulty in transferring patients to sites that were actually working caused more deaths and mismanagement of cadavers, which led to another public health crisis associated with the proliferation of the dead. One nurse stated:

There was a patient who died and spent three days in a corridor because it was not established where that patient was going to be taken if he died during the emergency. Since the hospital did not have electricity, you could not enter the morgue. (“Karla”, a nurse who works in a hospital)

The management of cadavers and their classification in the aftermath of Maria was worrying for participants, as providers received no prior instructions regarding how to respond to such a crisis. For example, “Juan”, a psychologist, observed:

I wouldn’t want to have been a doctor filling out a death certificate during the Hurricane. Causes of death? Well, a respiratory arrest. But where do I report that it was due to lack of electricity to power a breathing machine? Where do I report that I did not have asthma therapy and an asthmatic patient died? Where do I report that I did not have an EpiPen and an allergic person died? That did not appear in the certificate.

This participant viewed the lack of proper accounting for causes of mortality as a challenge to provide services to the very end, as it did not allow providers to properly name the immediate source of lethal health outcomes. Participants also described the implications of working under these extreme conditions on their colleagues. “Ricardo”, a health care administrator of a clinic in the rural area in San Juan, shared what happened at his hospital:

The psychologists had to do several group therapy sessions with the nurses to intervene and keep their spirits up as they were working overtime. We had nurses sleeping in the hospital. There were several crises with people… and also providers who bathed and slept in the hospital and ended up moving their clothes [to the facility] because there was no way out from the hospital.

Quotes like these present a post-Maria scenario where health professionals and administrators had very little room for effective action, as they were burdened by highly demanding circumstances and a convergence of health delivery challenges. Still, despite the health care system’s inefficiencies and disruptions, various manifestations of resilience were described by participants. One of them emerged from health care providers who began working independently with local communities, as reflected by one of our ethnographic observations.

In September 2018, the research team conducted an ethnographic visit to the Health and Acupuncture for the People Project (HAPP), one of the most prominent examples of creative strategies for health care provision the team identified in the study. HAPP is a self-funded community-based initiative led by “Esteban”, who was trained in Chinese Medicine and Acupuncture in New York and had provided services to communities in need for 4 years before the event. Upon seeing the collapse of the formal health care system, just 1 week after Hurricane Maria hit, Esteban’s group began working on the streets and public areas to provide free acupuncture services. As Esteban reported:

It took us a week and a half to organize ourselves to do the first Post-Maria clinic. […] When people found out that we were going to do the clinic, groups joined us because we announced it. A group of masseurs joined us, chiropractors joined us, people who do other types of modalities such as Reiki joined us.

Shortly after conducting these post-Maria clinics in San Juan, they quickly began providing itinerant acupuncture services throughout the Island, motivated primarily by “frustration by political and bureaucratic barriers” and their desire “to reach people who were abandoned by the traditional health care system”, as explained by Esteban himself.

The ethnographic visit referred to was to one of the clinics that HAPP carried out in the immediate post-storm period at the community-based organization *la Casa de la Cultura Ruth Hernández* (House of Culture Ruth Hernández), located in a sector of Puerto Rico’s capital city that showed evident infrastructure deterioration. One member of the research team who resided on the Island during Hurricane Maria was able to see first-hand the dynamics in the provision of services and the interactions between Esteban, the other auricular acupuncture providers, and the participants [see Additional file 1 - Excerpt of the ethnographic note taken during the visit to HAPP's clinic].

In conversations with Esteban and through a review of their records, it was calculated that during the months that followed Maria, HAPP provided free services to almost 350 persons between the ages of 11 and 87, most of whom had mental health and pain-related conditions. Esteban and his staff were delighted since 55% of patients reported that their conditions improved due to acupuncture, and these individuals often had nowhere else to turn to alleviate their symptoms following the storm.

Another example of resilience in health care provision was described by “Carlos”, a physician who administered a clinic and described how they managed to provide services in unusual settings so as to be responsive to patients’ needs.

Well, I think that we are very innovative. […] For example, we contacted *Farmacia Caridad* (a local community pharmacy), where the patients were looking for the medicines. It occurred to us that we should place a physician there who would receive the patients that arrived and attend to any medical situation. And things like this happened because, as a team, we thought about finding solutions and we were committed. I believe that without the commitment of the employees to help our patients and provide services, we would not have achieved anything.

## Discussion

The FHDR states that the “US is not prepared for large-scale natural disaster” such as Hurricane Maria. However, it is important to recognize that the reasons for this lack of preparedness should be understood in light of the particular context and history of the country, rather than a myopic focus on the storm itself. In order to understand why Puerto Rico, in particular, was not prepared to manage the challenges imposed by Hurricane Maria and why this natural event caused the collapse of the health care system (as described by participants themselves), the Island’s social, political, and economic situation must be taken into account.

The study findings suggest that health care providers and administrators interpreted the “collapse” of the health care system after Hurricane Maria as symptomatic of larger problems that had manifested on the Island long before the storm. What was experienced as an “entire collapse” was associated, on the one hand, with the rampant deterioration of the Island’s physical infrastructure (e.g., the power grid, roads, buildings, communication systems), and on a crisis of political and economic divestment in disaster preparedness, on the other. Regarding the former, infrastructural failures are closely linked to decades of neoliberal policies that have fostered the privatization of essential services as a means of managing the country’s economic crisis. Regarding the latter, participants associated the collapse with a poor local governance (e.g., lack of protocols and preparedness plans) and second-class treatment by the federal government (e.g., a hospital ship with suffocating protocols, imported physicians who did not speak Spanish and lacked knowledge of the local setting). It is significant that the latter political challenges were described by multiple participants as a form of “abandonment,” echoing what has been described in the published literature as colonial neglect [32]. In this sense, Hurricane Maria brought the Island's state of economic and political neglect into sharper view [33].

It was clear that the health care providers and administrators were confronted with an unprecedented catastrophic event resulting in an enormous cost in terms of human lives and suffering [25]. Echoing the tenets of the FHDR [26], almost all of Maria's deaths were chronically ill people, not only because of the event itself but as a consequence of systemic problems in the provision of disaster health care. While it may seem self-evident that extreme events threaten critical infrastructure, including power sources, many interlinked systems, including major components of the health care system (e.g., hospitals, life-sustaining equipment), depend on a reliable power grid to function effectively. In Puerto Rico, it has been well-established that the interruption of medical care due to the prolonged power outage was the primary cause of sustained high mortality rates in the months after the Hurricane [25]. The high death toll brought into relief the local and federal governments’ lack of preparedness and inadequate response. The mismanagement of the dead (and the lack of transparency in the actual death toll) also accentuated a deeply rooted sense of "mistrust," as described by one participant. In fact, the mistrust was not only linked to the government’s inability to deal effectively with disaster management, but it also emanated from resentment over its perceived sense of “alienation” from the population’s suffering. Certainly, this lack of trust could be linked to the local population's realization, almost immediately after the event, that the Island’s future was jeopardized primarily by factors that transcended the hurricane itself: politics, the economic crisis, and the effects of long-term austerity [34, 35]. The latter has been notably exacerbated since the advent of *la Junta* [36].

Finally, participants detailed the convergence of challenges they faced in order to provide services to the population. Still, amidst these difficulties, important examples of community resilience are evident. Importantly, the clearest examples of resilience this study uncovered were not those of formal institutions and bureaucracies but rather resulted from committed local actors and networks who took the crisis into their own hands. Examples of such community responses echo the FHDR call for grassroot and community-based initiatives to strengthen the health sector in order to manage mass medical care needs in post-disaster contexts [26]. The examples provided by participants included the transformation and repurposing of health care sites to expand services (e.g., turning a pharmacy into a clinic) and the direct involvement of health professionals with communities in order to surpass bureaucratic difficulties when providing in post-disaster aid.

The HAPP initiative is an emblematic case of resilience since it represents a collaborative effort between health professionals and community members focused on complementary medicine, which existed at the margin of the traditional biomedical model of health care provision. These characteristics help explain why HAPP was more agile in adapting to the communities’ needs in Maria’s aftermath. In addition, strengthened by a structure of volunteer workers and offering free services, they were able to sidestep many of the bureaucratic barriers of the formal health care system, emphasizing instead the provision of care, including treatment for stress, anxiety, and pain. Initiatives like this highlight that in order to address disaster-related challenges, the health sector needs to be more robust and connected to preparedness and resilience initiatives in local communities, a central idea of the FHDR. In this sense, the commitment of these health professionals to provide care to those most in need during extremely challenging circumstances was an example of how health care system functionality could be maintained and therefore meet the needs of the most vulnerable sectors.

There is a growing criticism of the concept of resilience that is based on its being utilized as a neoliberal strategy to hold individuals in settings such as Puerto Rico responsible for creating the conditions to survive and stay healthy [33], independent of the deteriorating context in which they live. Therefore, it is important to examine efforts that arise organically from the collaboration of communities and professional groups (e.g., medical brigades) for the benefit of public health. Certainly, post-disaster contexts require solidarity aimed at meeting the health needs of communities when conditions do not allow access to traditional biomedical services. These grassroots responses are manifestations of resilience that seem very distant from neoliberal approaches to health care delivery that dominate many discussions of disaster resilience. The challenge is to find ways to support these initiatives without burdening them with the excessive bureaucracy of the traditional health care system, particularly in the context of systemic collapse.

The data shared in this article should be contextualized in light of this study’s limitations. The qualitative techniques used in this study are not intended to portray a representative assessment of the perspectives of all health professionals in Puerto Rico. Therefore, no generalizations about the frequency of particular perceptions or experiences concerning the health care system's collapse should be made. Also, future studies should include representatives of other important disciplines associated with the provision of health care services, such as social workers and counselors. Future research should take these limitations into considerations when exploring the implications of natural disasters on Puerto Rico’s health care system.

## Conclusions

This study’s results highlight that health care providers and administrators perceived the collapse of Puerto Rico’s health care system as the culmination of a process of a gradual deterioration of the Island's infrastructure due to broader political and economic forces. Critical failures in the Island's health care system were primarily responsible for the high number of deaths related to Hurricane Maria. The local and federal government response to the collapse of the health care system was understood as inadequate, fostering a sense of abandonment or neglect amongst frontline health care workers. This natural phenomenon unveiled the multiple difficulties faced by Puerto Rico in general, and the health sector specifically, which place it in a position of extreme vulnerability to natural disasters in the future. However, Hurricane Maria also unveiled the commitment and solidarity of health professionals who sought innovative ways to meet the health needs of communities in times of crisis. The initiatives shared in this analysis are important examples of how community resilience should be considered part of the health sector's disaster management strategy. In a setting like Puerto Rico, where structural difficulties are constant, and the threat of natural disasters is inevitable, it is crucial to take into consideration the perspective of health care professionals and administrators in order to develop a culture of disaster resilience in which they can implement innovative and agile strategies to overcome the limitations of the current model of provision of health care services when the next disaster strikes.

## Supplementary Information

**Additional file 1**. Excerpt of the ethnographic note taken during the visit to HAPP's clinic.

## Data Availability

This publication contains a minimal data set within the manuscript. All relevant data are within the manuscript. Transcripts cannot be shared publicly as participants did not provided consent for it. People interested in accessing the transcripts can contact the Florida International University's Institutional Review Board in order for them to evaluate researchers who meet the criteria to access confidential data (contact via email at mdemelen@fiu.edu). Transcripts are in the Spanish language.

## Abbreviations

NIH: National Institutes of Health of the United States of America
US: United States
SSI: Semi-structured interviews
HAPP: Health and Acupuncture for the People Project
FHDR: Framework for Healthcare Disaster Resilience
