# Supplementary material for: The impact of Hurricane Maria on Puerto Rico’s health system: post-disaster perceptions and experiences of health care providers and administrators
Source: Glob Health Res Policy. 2021 Nov 10;6:44. doi: 10.1186/s41256-021-00228-w (PMC8577961; doi:10.1186/s41256-021-00228-w)
Supplement: Supplementary file 1 — Additional file 1. Excerpt of the ethnographic note taken during the visit to HAPP's clinic. [file 41256_2021_228_MOESM1_ESM.docx]

**Excerpt of an ethnographic note taken during a visit to the Health**

**and Acupuncture for the People Project’s post-disaster clinic**

This time [the mobile clinic] was located in Río Piedras, one of the main sectors of the metropolitan area of San Juan; specifically, at *Casa de la Cultura Ruth Hernández Torres*. The cultural house was very active in sharp contrast to its surroundings. The house was well kept and maintained by the community, while the surrounding buildings are either abandoned or completely dilapidated. The buildings reflect a mix of the economic crisis on the Island and the Hurricane's still visible effects. You could spot the stairs in front of the town church from the house, which were full of homeless people who were visibly injecting drugs.

Upon entering the cultural house, I noticed that the [HAPP] group had rearranged the seats in order to form semicircles in which people could see each other and the facilitator. It was evident that some of them had been there before, as they chatted amongst themselves about how they were doing in terms of their health. I counted a total of 45 people that were there to receive services. Ten of them were there for the first time, as evidenced by Esteban’s interactions with them. The age range surprised me as there was a balanced mix of older (in their 50s and 60s) and younger (in their 20s) people. I was not expecting to see so many young people there, which evidenced the impact of Hurricane Maria and the economic crisis on all age groups.

The chatter was interrupted by Esteban’s tone of voice, which I would describe as simultaneously warm and directive. He explained the nature of the group, the health services they provided, the conditions they treated, and how those that were there for the first time would need to sign a release. He stressed that the services provided were free of charge and that donations and the volunteers covered the costs. Esteban then went on to explain their acupuncture protocol in detail. He focused on the scientific data backing the protocol, which primarily addressed anxiety and stress. He also mentioned its usefulness for pain management and sleep problems. Esteban stressed that the group used sterile needles for everyone. He also repeated on several occasions that HAPP did this on a voluntary basis. A phone rang, and Esteban raised his voice to tell people to shut them off. “This is a space of relaxation,” he mentioned once again in what seemed more like a scolding than a reminder. Esteban went on to explain that massages were available for people who completed the acupuncture protocol. “Let’s allow people who did not get them last week to do so today”, he announced. “Let’s share the resource and not do what the government does.” People chuckled at his criticism of the government. Everyone there seemed to know that Esteban was making reference to the resources, like the water bottles that appeared expired in an airstrip in Ceiba, which the government failed to share amongst those affected by the Hurricane. With that orientation completed, Esteban and a team of three other individuals began placing needles in people’s ears.

Once everyone received the basic acupuncture protocol, Esteban went around the group asking about particular concerns or conditions that they needed to treat. He then used needles on their hands, feet, head, and shoulders, as needed. As this different process took place, other people were done with their treatment, and their needles were removed (the process took 30 minutes). Seeds were then placed in the same pressure point in their ears so that they could manually self-stimulate these areas throughout the week. Finally, they received a piece of cotton with essential oils as a form of aromatherapy. Those that could not make it to the massage table left the cultural house, and one could hear them say to others, “see you next week,” as they walked out of the building and back onto the streets of Río Piedras.

Two things seemed particularly important to me in this ethnographic visit. First, the group of health professionals was able to keep their operation running while most other clinics around them were closed. They were not burdened by the administrative bureaucracy of the health care system, and this allowed them to provide services. Second, they seemed keenly aware that people felt abandoned by the usual sites where they would receive services, and the group made a conscious effort to validate those feelings, and yet imbue them with hope that the crisis would eventually pass. In the meantime, community resilience, supported by these health professionals, seemed to be the rallying cry to organize patients and providers.
